# Prevalence of sarcopenia in older women and level of agreement between the diagnostic instruments proposed by the European Working Group on Sarcopenia in Older People 2 (EWGSOP2)

**DOI:** 10.1186/s12891-023-06287-z

**Published:** 2023-03-11

**Authors:** Daiana Vieira Sutil, Adriana Netto Parentoni, Leonardo Augusto Da Costa Teixeira, Bruno de Souza Moreira, Amanda Aparecida Oliveira Leopoldino, Vanessa Amaral Mendonça, Ana Cristina Rodrigues Lacerda, Ana Lúcia Danielewicz, Núbia Carelli Pereira de Avelar

**Affiliations:** 1 Universidade Federal de Santa Catarina (Federal University of Santa Catarina), Araranguá, SC Brazil; 2 Universidade Federal dos Vales do Jequitinhonha e Mucuri (Federal University of the Jequitinhonha and Mucuri Valleys), Diamantina, MG Brazil; 3 Núcleo de Estudos em Saúde Pública e Envelhecimento (Center for Studies in Public Health and Aging), Universidade Federal de Minas Gerais e Fundação Oswaldo Cruz - Minas Gerais (Federal University of Minas Gerais and Oswaldo Cruz Foundation - Minas Gerais), Belo Horizonte, MG Brazil; 4 Faculdade Ciências Médicas de Minas Gerais (Faculty of Medical Sciences of Minas Gerais), Belo Horizonte, MG Brazil; 5 Department of Health Sciences, Universidade Federal de Santa Catarina (Federal University of Santa Catarina), 3201 Jardim das Avenidas – Araranguá, CEP: 88.906-072 Araranguá, SC Brazil

**Keywords:** Sarcopenia, Aged, Prevalence, Diagnosis, EWGSOP2

## Abstract

**Background:**

The European Working Group on Sarcopenia in Older People 2 (EWGSOP2) proposed the use of different diagnostic tools to assess sarcopenia. This study aimed to determine prevalence rates of sarcopenia according to the diagnostic instruments proposed by EWGSOP2 and to assess their level of agreement in older Brazilian women.

**Methods:**

A cross-sectional study with 161 community-dwelling older Brazilian women. Probable sarcopenia was assessed through Handgrip Strength (HGS) and the 5-times sit-to-stand test (5XSST). In addition to reduced strength, Appendicular Skeletal Muscle Mass (ASM) (obtained by Dual-energy X-ray absorptiometry) and ASM/height² were considered for diagnosis confirmation. Sarcopenia severity was determined by reduced muscle strength and mass and poor functional performance assessed by Gait Speed (GS), Short Physical Performance Battery (SPPB), and Timed Up and Go test (TUG). McNemar’s test and Cochran’s Q-test were used to compare sarcopenia prevalence. Cohen’s Kappa and Fleiss’s Kappa tests were used to assess the level of agreement.

**Results:**

The prevalence of probable sarcopenia was significantly different (p < 0.05) when using HGS (12.8%) and 5XSST (40.6%). Regarding confirmed sarcopenia, the prevalence was lower when using ASM/height² than with ASM. Regarding severity, the use of SPPB resulted in a higher prevalence in relation to GS and TUG.

**Conclusion:**

There were differences in the prevalence rates of sarcopenia and low agreement between the diagnostic instruments proposed by the EWGSOP2. The findings suggest that these issues must be considered in the discussion on the concept and assessment of sarcopenia, which could ultimately help to better identify patients with this disease in different populations.

## Introduction

Sarcopenia is a disease (ICD-10-MC) diagnosed by a reduction in the quality and/or quantity of muscle mass, which occurs due to gradual and generalized muscle changes [1]. Furthermore, the European Working Group on Sarcopenia in Older People 2 (EWGSOP2) defined that muscle strength reduction should be considered the first stage in screening for this condition [1]. For diagnostic confirmation, in addition to the reduction in muscle strength, reduced muscle quantity and/or quality should be observed. The severity of sarcopenia would then be defined by changes in strength, muscle quantity/quality, and poor functional performance assessed by Gait Speed ​​(GS), Short Physical Performance Battery (SPPB), or Timed Up and Go test (TUG) [1].

Recent estimates suggest that the overall prevalence of sarcopenia in older adults can range from 10.0% [2] to 82.1% [3, 4]. In addition, previous studies show that the prevalence of sarcopenia is higher in older women than in older men [5–7]. A possible explanation for the higher prevalence in women may be related to hormonal aspects and less muscle mass [8, 9]. Additionally, women present cumulative disadvantages throughout life, including poor access to education, income, and food, which consequently leads to a greater likelihood of poverty and, therefore, greater health problems and disabilities in old age [10].

Evidence suggests that the prevalence of sarcopenia can vary depending on the diagnostic algorithm, such as EWGSOP1, EWGSOP2, Asia Working Group for Sarcopenia (AWGS), International Working Group on Sarcopenia (IWGS), and Foundation for the National Institutes of Health (FNIH) [11–18]. Recently, Anand et al. (2022) demonstrated weak agreement between diagnostic criteria for sarcopenia when using different diagnostic algorithms [3].

Although the literature reports a lack of agreement between different algorithms [3, 11–18], to our knowledge, no previous study has investigated the differences in the prevalence of sarcopenia and the level of agreement between the diagnostic instruments proposed within the same consensus. The most current recommendation on sarcopenia is from EWGSOP2, which suggests strategies for screening (probable sarcopenia), diagnostic confirmation (confirmed sarcopenia), and severity of the disease. It is expected that the findings of this study provide guidance to health professionals and public managers on the choice of instruments used for sarcopenia diagnosis, which is essential to the planning of health service actions, such as the establishment of preventive approaches and therapeutic strategies for this condition. Thus, the aims of this study were to determine the prevalence rates of sarcopenia in older women according to the diagnostic instruments proposed by EWGSOP2 and to assess their level of agreement.

## Materials and methods

### Study design

This was a cross-sectional study conducted with community-dwelling older women, which was approved by the Research Ethics Committee of the Universidade Federal dos Vales do Jequitinhonha e Mucuri (Federal University of the Jequitinhonha and Mucuri Valleys) (protocol no. 1.461.306), following the principles described in the Declaration of Helsinki.

### Eligibility criteria

Older women aged 65 years and over residing in the community and able to walk independently were included. The exclusion criteria were: (a) younger than 65 years old; (b) cognitive decline detectable by the Mini-Mental State Examination, considering the Brazilian cutoff points related to schooling, proposed by Bertolucci et al. [19]: 13 points for illiterates; 18 points for people with 1 to 7 years of schooling; 26 points for those with 8 years or more of schooling; (c) neurological sequelae that could interfere in the results of the tests proposed by EWSGOP2 [Handgrip Strength (HGS), 5-times sit-to-stand test (5XSST), GS, SPPB, and TUG]; (d) hospitalization in the last three months; (e) fractures in the lower limbs for less than six months and with orthopedic problems; (f) musculoskeletal, respiratory, cardiovascular, and thyroid diseases or other inflammatory diseases in the acute phase; (g) practicing physical activity on a regular basis (at least three times a week); (h) presence of metal in their bodies; (i) visual or hearing impairment; or (j) bedridden.

### Procedures

Participants were selected for convenience and recruited through calls, invitations, and announcements in Basic Health Units, public places, and a geriatric office in Diamantina, Minas Gerais, Brazil. The older women were asked about the eligibility criteria of the study, as well as use of medication, history of falls in the last 6 months, and level of physical activity. All participants signed an informed consent form.

Data were collected between June 2016 and June 2017 by trained healthcare professionals at the Exercise Physiology Laboratory of the Universidade Federal dos Vales do Jequitinhonha e Mucuri. The evaluators who performed the Appendicular Skeletal Muscle Mass (ASM) measurements using Dual-energy X-ray absorptiometry (DXA) were different from those who applied the functional tests. Initially, the women were submitted to an anthropometric evaluation (body mass and height) and then the ASM. Both assessments were performed under fasting conditions. Subsequently, the functional tests were performed: HGS, 5XSST, SPPB, GS, and TUG. The sequence of execution of the functional tests was randomly determined.

### Instruments

#### Instruments for diagnosing probable sarcopenia

To screen for probable sarcopenia, the HGS and 5XSST were used.

##### HGS

The participants performed an isometric contraction applied on the Jamar® hand dynamometer, in a sitting position, with shoulder and wrist in a neutral position and elbow at 90 degrees of flexion [20]. Three measurements were performed with the dominant hand and the highest value among the three measurements was used in the analyses. A value ​​lower than 16kgf is indicative of probable sarcopenia [1].

##### 5XSST

To perform the test, the time taken by the participants to rise from and sit on a chair five times, as fast as possible, with the upper limbs crossed over the chest, was recorded [21]. Taking more than 15 s to perform the test is indicative of probable sarcopenia [1].

#### Instruments for confirming the sarcopenia diagnosis

In addition to the reduction in muscle strength assessed by HGS or 5XSST, it is necessary to assess the ASM using DXA (Lunar Radiation Corporation, Madison, Wisconsin, USA, DPX model) to confirm the sarcopenia diagnosis.

For ASM measurement, the participants had to wear light clothes and not have metallic objects in or on their bodies. They were positioned in the scanning area of ​​the equipment so that the sagittal line passed through the center of anatomical points such as the skull, spine, pelvis, and legs. For optimal positioning, Velcro bands joined the legs, knees, and feet. Data on lean and fat muscle mass were collected. Data adjusted for height (ASM/height²) were also obtained. The presence of sarcopenia was confirmed when ASM and ASM/height^2^ were lower than 15 kg and 5.5 kg/m^2^, respectively [1].

#### Instruments for assessing sarcopenia severity

To assess sarcopenia severity, participants with confirmed sarcopenia performed three functional tests: Gait Speed (GS), Short Physical Performance Battery (SPPB), and Timed Up and Go test (TUG).

To assess GS, the participants walked a four-meter distance at a comfortable/habitual pace. Timing started when one of the feet crossed the starting line and ended when one of the feet completely crossed the finish line [22]. GS (m/s) was obtained by dividing the distance traveled (m) by the time (s). A GS lower than or equal to 0.8 m/s is indicative of severe sarcopenia [1].

The SPPB is a battery of tests used to objectively assess lower limb function in older adults through three tests: static body balance, lower limb muscle strength, and gait. For each of the tests, scores range from 0 to 4 points, with a maximum score on the instrument of 12 points. The higher the score, the better the performance [23]. A score less than or equal to 8 is indicative of severe sarcopenia [1].

The TUG is a test that consists of recording the time required by the individual to get up from a chair, walk three meters, pivot around an obstacle, return, and sit down again. The longer the time to perform the test, the worse the functional performance [24]. A time greater than or equal to 20 s is indicative of severe sarcopenia [1].

### Sample calculation

The sample calculation was performed considering the sarcopenia prevalence of 4.6% in older Brazilian women using the EWGSOP2 criteria [25]. Assuming an absolute precision of 5% and a confidence interval (CI) of 95%, a minimum sample size of 68 participants would be necessary to carry out the present study.

### Statistical analysis

Data were entered into SPSS software (IBM®, Chicago, IL, USA), version 23.0. The significance level adopted for the analyses was 0.05. Prevalence was described using relative frequency (%). To compare the prevalence of sarcopenia between the different diagnostic instruments, McNemar’s test (probable and confirmed sarcopenia) and Cochran’s Q-test (severe sarcopenia) were used. To assess the level of agreement between the diagnostic tools for sarcopenia, Cohen’s Kappa test (probable and confirmed sarcopenia) and Fleiss’s Kappa test (severe sarcopenia) were used. To interpret the agreement analysis, the classification categories proposed by McHugh (2012) were considered [26]: 0 to 0.20 represents no agreement; 0.21 to 0.39 represents minimal agreement; 0.40 to 0.59 represents weak agreement; 0.60 to 0.79 represents moderate agreement; 0.80 to 0.90 represents strong agreement; and above 0.90 represents almost perfect agreement.

## Results

Of the 337 older women initially contacted, 33 were younger than 65 years old and 76 refused to participate. Of the older women who signed an informed consent form, 13 reported having thyroid deficiency, 8 presented decompensated lung disease, 2 had hearing impairment, 2 had visual impairment, 7 had orthopedic problems, 10 presented cognitive decline identified by the Mini-Mental State Examination, 7 were bedridden, 13 practiced physical activity on a regular basis, 2 had been recently hospitalized, and 3 had metal in their bodies. This left a total of 161 eligible participants.

The 161 participants were community-dwelling older women (age: 74.4 ± 7.3 years; body mass: 61.0 ± 10.9 kg; height: 1.5 ± 0.1 m; BMI: 27.1 ± 4.6 kg/m^2^). They used, on average, 3.4 (± 2.1) medications, and 21.7% had a history of falls in the last 6 months.

**Fig. 1:**
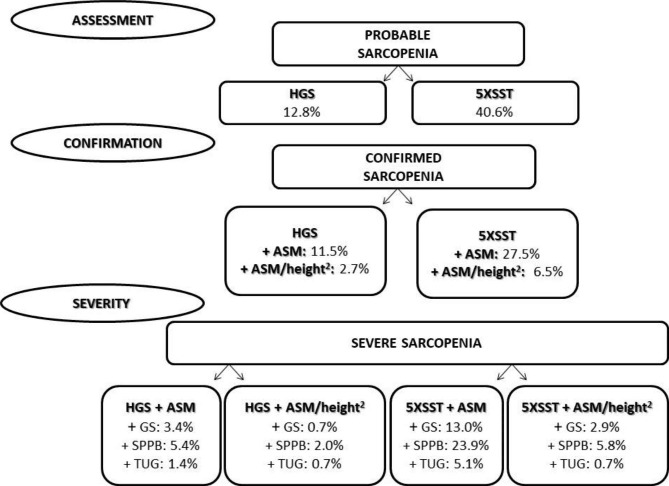
EWGSOP2 algorithm for case-finding, making a diagnosis, and quantifying severity in practice, and prevalence rates. **Note**: HGS: Handgrip Strength; 5XSST: 5-times sit-to-stand test; ASM: Appendicular Skeletal Muscle Mass; GS: Gait Speed; SPPB: Short Physical Performance Battery; TUG: Timed Up and Go test

### Probable sarcopenia

**Fig. 2:**
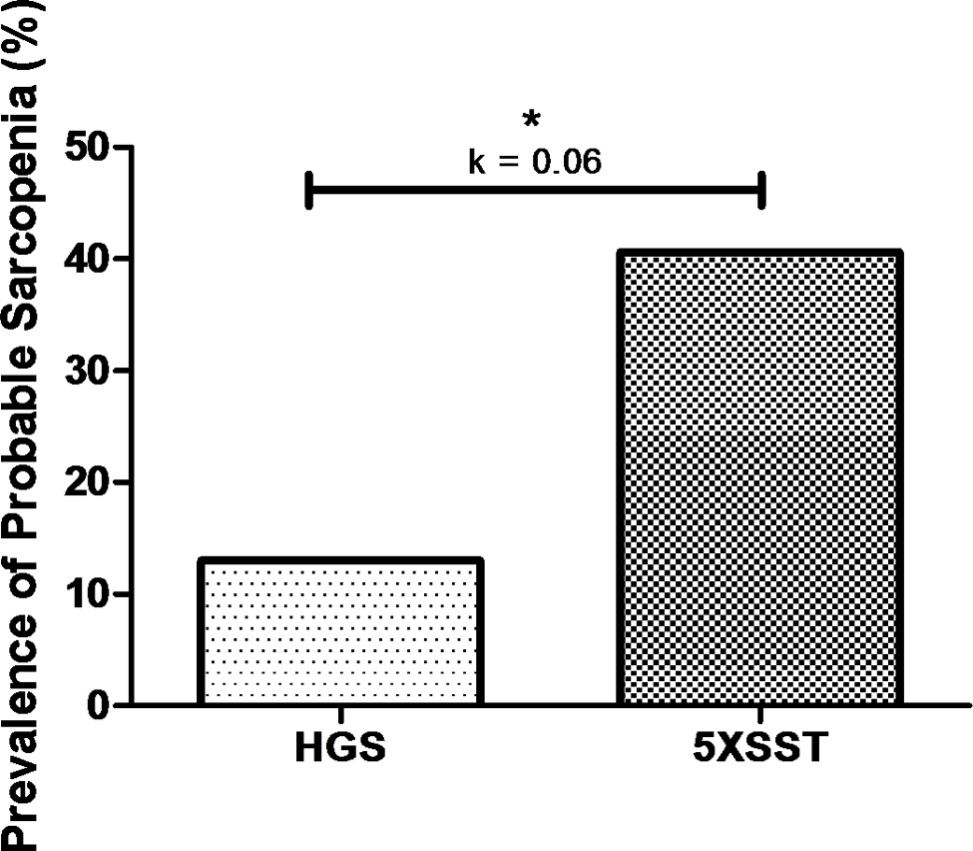
Prevalence of probable sarcopenia (%) and agreement between diagnostic instruments. * Statistically significant difference in the prevalence of probable sarcopenia in older women between the diagnostic instruments. **Note**: HGS: Handgrip Strength; 5XSST: 5-times sit-to-stand test. Agreement analysis: K = 0–0.20: no agreement; K = 0.21–0.39: minimal agreement; K = 0.40–0.59: weak agreement; K = 0.60–0.79: moderate agreement; K = 0.80–0.90: strong agreement; K > 0.90: almost perfect agreement

The prevalence rates of probable sarcopenia were 12.8% and 40.6%, assessed through HGS and 5XSST, respectively (Fig. 1). There was a statistically significant difference in the prevalence of probable sarcopenia between the diagnostic instruments (X^2^ = 23.56; p < 0.01). Cohen’s Kappa test showed a lack of agreement between these diagnostic instruments [K = 0.06; p = 0.34] (Fig. 2).

### Confirmed sarcopenia

**Fig. 3:**
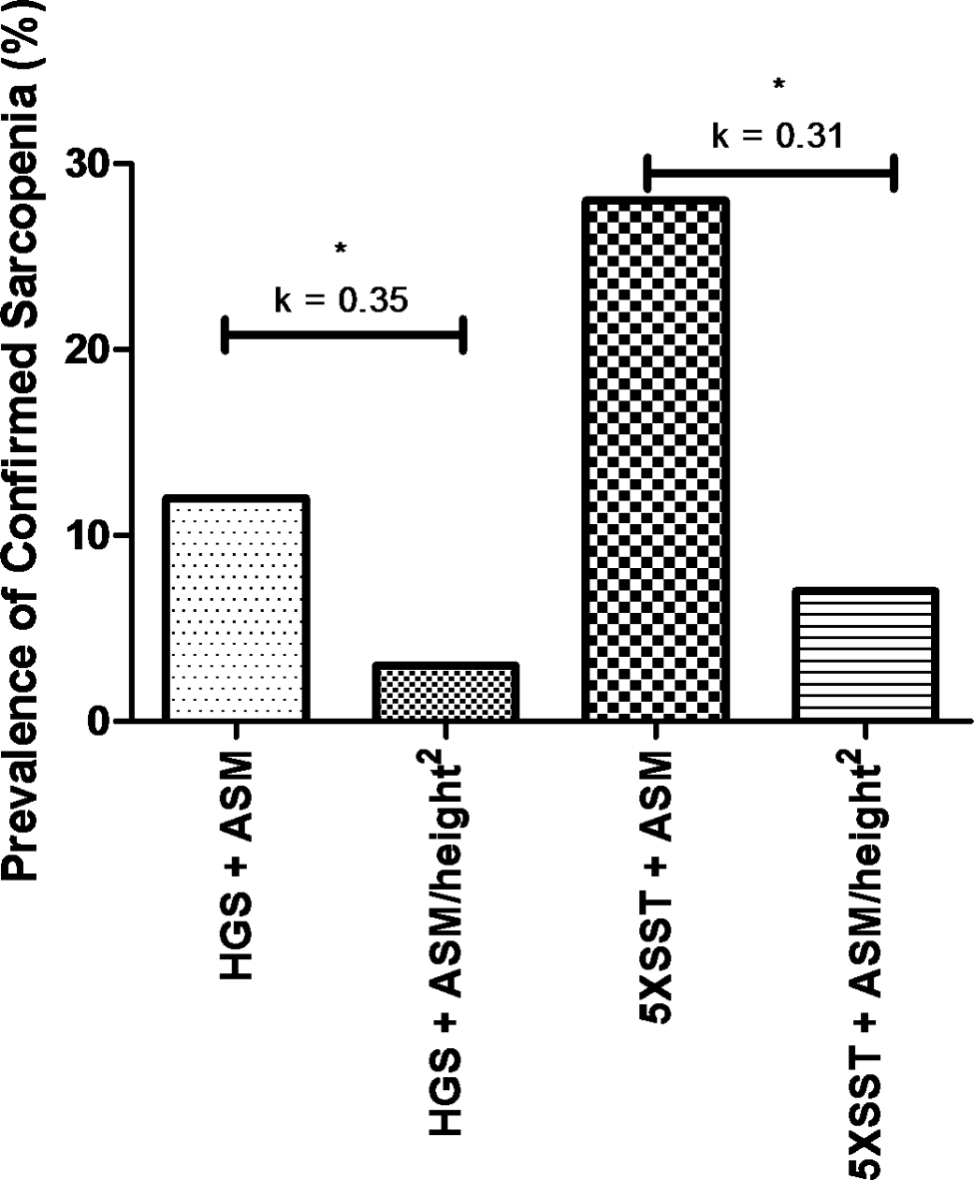
Prevalence of confirmed sarcopenia (%) and agreement between diagnostic instruments. * Statistically significant difference in the prevalence of confirmed sarcopenia in older women between the diagnostic instruments. **Note**: HGS: Handgrip Strength; ASM: Appendicular Skeletal Muscle Mass; 5XSST: 5-times sit-to-stand test. **Agreement analysis**: K = 0–0.20: no agreement; K = 0.21–0.39: minimal agreement; K = 0.40–0.59: weak agreement; K = 0.60–0.79: moderate agreement; K = 0.80–0.90: strong agreement; K > 0.90: almost perfect agreement

The prevalence rates of confirmed sarcopenia were 11.5% and 2.7%, using HGS + ASM and HGS + ASM/height^2^, respectively (Fig. 1). There was a statistically significant difference in the prevalence of confirmed sarcopenia between the diagnostic instruments (X^2^ = 11.08; p < 0.01). Cohen’s Kappa test showed minimal agreement between these diagnostic instruments [K = 0.35; p < 0.01] (Fig. 3).

The prevalence rates of confirmed sarcopenia were 27.5% and 6.5%, using 5XSST + ASM and 5XSST + ASM/height^2^, respectively (Fig. 1). A statistically significant difference was also observed in the prevalence of confirmed sarcopenia between these diagnostic instruments (X^2^ = 27.03; p < 0.01). Cohen’s Kappa test showed minimal agreement between these diagnostic instruments [K = 0.31; p < 0.01] (Fig. 3).

### Severe sarcopenia

**Fig. 4:**
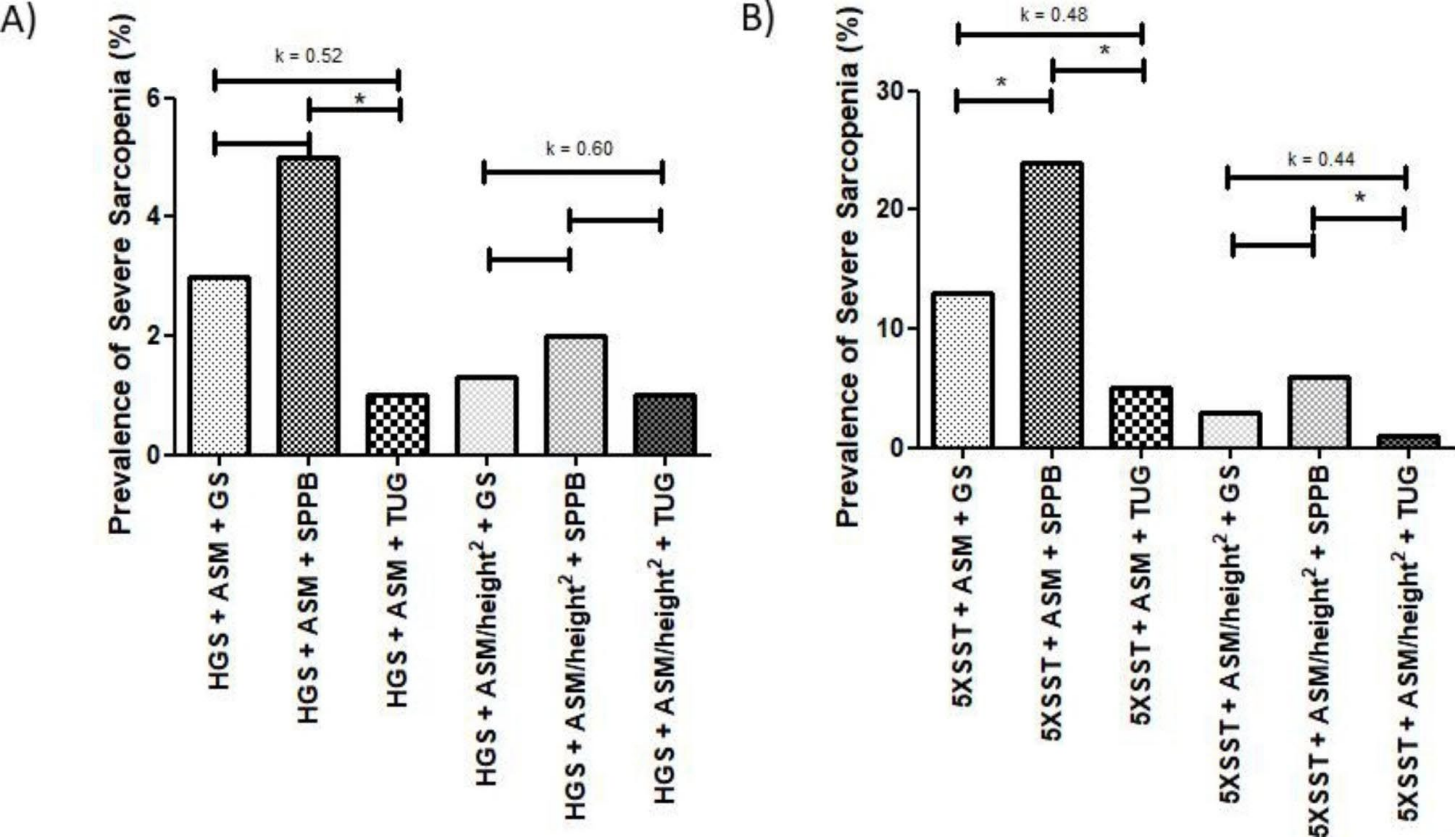
Prevalence of severe sarcopenia (%) and agreement between diagnostic instruments. * Statistically significant difference in the prevalence of severe sarcopenia in older women between the diagnostic instruments. **Note**: HGS: Handgrip Strength; ASM: Appendicular Skeletal Muscle Mass; GS: Gait Speed; SPPB: Short Physical Performance Battery; TUG: Timed Up and Go test; 5XSST: 5-times sit-to-stand test. **Agreement analysis**: K = 0–0.20: no agreement; K = 0.21–0.39: minimal agreement; K = 0.40–0.59: weak agreement; K = 0.60–0.79: moderate agreement; K = 0.80–0.90: strong agreement; K > 0.90: almost perfect agreement

The prevalence rates of severe sarcopenia were 3.4%, 5.4%, and 1.4%, using HGS + ASM + GS, SPPB, or TUG, respectively (Fig. 1). Cochran’s Q-test showed a statistically significant difference in the prevalence of severe sarcopenia between the three diagnostic instruments (X^2^_(2)_ = 7.71; p = 0.02). Pairwise comparisons showed that the prevalence of severe sarcopenia was significantly higher when using SPPB than with TUG (p = 0.02). Fleiss’s Kappa test showed weak agreement between the three diagnostic instruments [K = 0.52, p < 0.01] (Fig. 4A).

When using HGS + ASM/height^2^ + GS, SPPB, or TUG, the prevalence rates of severe sarcopenia were 0.7%, 2.0%, and 0.7%, respectively (Fig. 1). There was no statistically significant difference in the prevalence of severe sarcopenia between the three diagnostic instruments (X^2^_(2)_ = 4.00; p = 0.135). Fleiss’s Kappa test showed moderate agreement between the three diagnostic instruments [K = 0.60, p < 0.001] (Fig. 4A).

The prevalence rates of severe sarcopenia were 13.0%, 23.9%, and 5.1%, using the 5XSST + ASM + GS, SPPB, or TUG, respectively (Fig. 1). Cochran’s Q-test showed a statistically significant difference in the prevalence of severe sarcopenia between the three diagnostic instruments (X^2^_(2)_ = 39.31; p < 0.01). Pairwise comparisons showed that the prevalence of severe sarcopenia was significantly higher when using SPPB than with GS (p < 0.01) and TUG (p < 0.01). Fleiss’s Kappa test showed weak agreement between the three diagnostic instruments [K = 0.48, p < 0.01] (Fig. 4B).

When using 5XSST + ASM/height^2^ + GS, SPPB, or TUG, the prevalence rates of severe sarcopenia were 2.9%, 5.8%, and 0.7%, respectively (Fig. 1). There was a statistically significant difference in the prevalence of severe sarcopenia between the three diagnostic instruments (X^2^_(2)_ = 10.57; p < 0.01). Pairwise comparisons showed that the prevalence of severe sarcopenia was significantly higher when using SPPB than with TUG (p = 0.004). Fleiss’s Kappa test showed weak agreement between the three diagnostic instruments [K = 0.44, p < 0.01] (Fig. 4B).

## Discussion

This study showed differences in the sarcopenia prevalence and severity tested using instruments proposed by the EWGSOP2 (rates: 0.7–40.6%). Moreover, the level of agreement between all the instruments evaluated by the Kappa test was, in general, minimal or weak.

Similar to the findings of the present study, recent previous evidence found differences in the prevalence of sarcopenia between several diagnostic criteria in older Asian adults, with rates ranging from 5.9 to 82.1% [3]. According to the authors, the great variability in prevalence rates is related to the several cutoff points existing in different diagnostic criteria for the definition of adequate muscle mass, which may vary between geographic regions and, therefore, must be adapted to the ethnic group to which it is being applied [3].

In this study, the prevalence of probable sarcopenia assessed by 5XSST was higher than when assessed by HGS and there was no agreement between the two screening tests. A possible explanation for the difference in prevalence between these two instruments may be related to the specificity of the assessment. While the HGS assesses upper limb muscle strength, the 5XSST assesses muscle strength in the lower limbs [27]. Moreover, tests such as the 5XSST may represent general physical performance and not only muscle strength [28].

Unlike the assessment using the 5XSST, when using the manual dynamometer, aspects of physical performance such as balance, endurance, and mobility are neglected. Corroborating this argument, a previous study by Felicio et al. (2014) found a low correlation between HGS and lower limb muscle performance in community-dwelling older women [29]. However, while the study by Felicio et al. (2014) evaluated the lower limbs using specific isokinetic tests [29], this study used physical-functional performance tests to provide a global evaluation. Furthermore, similar to the present findings, a previous study found a higher prevalence of probable sarcopenia when assessed using 5XSST (91.0%) compared with HGS (29.0%), suggesting that the assessment of lower limb muscles may be more sensitive to detecting loss of muscle strength in older adults [30]. Thus, muscle assessment of the lower limbs seems to be more adequate for the screening of sarcopenia. It may be the case that changes in lower limb muscles appear in earlier stages of the disease. However, this cannot be inferred from the findings of this cross-sectional study, although it may be an interesting topic for further longitudinal research.

The present study found a high prevalence of probable sarcopenia using 5XSST (40.6%). Other authors also found high prevalence rates in their sample when using this functional test. For example, de Souza et al. (2022) found a prevalence of probable sarcopenia of 64.1% for older women from the city of Balneário Arroio do Silva in the state of Santa Catarina, Brazil [31]. Another study conducted by de Souza et al. (2022) observed a prevalence of probable sarcopenia of 42.0% in older Brazilian women using data from a study with probabilistic sampling carried out in Florianópolis in the state of Santa Catarina, Brazil [32]. Swan, Warters and O’Sullivan (2022) observed that 26.1% of participants aged 60 years and over from the English Longitudinal Study of Ageing (ELSA) met the criteria for probable sarcopenia based on poor performance in 5XSST [33]. In addition, when examined for socioeconomic status, these authors found that the prevalence of probable sarcopenia was over 2-times higher in the most disadvantaged socioeconomic status group compared with the least disadvantaged (47.0% vs. 20.6%, respectively) [33]. Thus, divergences in the prevalence of probable sarcopenia across studies may also be related to the socioeconomic conditions of the populations studied. Notably, previous research by our group identified cutoff points for sociodemographic and anthropometric variables in screening for probable and confirmed sarcopenia in community-dwelling older adults [34]. In addition, Kim and Won (2019) found a high prevalence of confirmed sarcopenia in older Korean women (14.4%) when using 5XSST + ASM, which is in line with the present results (27.5%) [35]. Recently, Sayer and Cruz-Jentoft (2022) pointed out that studies on this topic need to be encouraged in low and middle-income countries to address local needs as well as for developing a global perspective on sarcopenia [36].

However, some restrictions regarding the use of HGS and 5XSST need to be highlighted. For patients with upper extremity impairment and/or affected by rheumatoid arthritis, hand osteoarthritis, or carpal tunnel syndrome, HGS may not be an accurate reflection of muscle strength and may lead to underestimations. Similarly, the 5XSST also has a restricted capacity to assess a wide variation in ability, which is relevant in older adults, since some cannot complete the five attempts and are therefore not assigned a score (floor effect). The utility of this test is therefore limited in individuals suffering from moderate to severe mobility limitations [37]. Despite the limitations of using these instruments to screen for probable sarcopenia, evidence suggests that HGS is accurate in detecting sarcopenia in community-dwelling older women [38].

In the present study, a higher prevalence of confirmed sarcopenia was observed when using ASM than with ASM/height². Bijlsma et al. (2013) also found that ASM is better for predicting physical performance in older adults than ASM/height² [39]. The authors argue that, when adjusted for height, ASM can underestimate sarcopenia in obese individuals and overestimate sarcopenia in underweight older adults [39]. Since this index is positively correlated with BMI, individuals with a greater BMI due to a larger amount of fat are less likely to be classified as having sarcopenia [39]. Furthermore, when comparing ASM adjusted for weight, body mass index, and height, Kim, Jang and Lim (2016) and Figueiredo et al. (2014) found a lower prevalence of sarcopenia using ASM/height² [40, 41]. It should be mentioned that in the present study, 47.8% of older women were classified as obese, which might partially explain the difference in the prevalence of sarcopenia between the two criteria. There is an ongoing debate about the best adjustment and whether the same method can be used for all populations [1, 40].

In this study, it was observed that the prevalence of severe sarcopenia detected by GS and TUG was 0.7% and by SPPB was 2.0%, considering HGS + ASM/height², showing no significant difference and exhibiting a moderate agreement between the diagnostic instruments. This similar prevalence may have occurred because only one participant had severe sarcopenia detectable by GS and TUG, while three participants were identified using SPPB. In consonance with our results, Paula et al. (2016) found a moderate-to-high agreement when using GS and TUG in their sample of older Brazilian women [42]. According to these authors, small changes in physiological capacity can be noted in a similar way when using these two physical-functional performance tests.

In the current study, the lowest prevalence of severe sarcopenia using 5XSST + ASM/height^2^ was found when the TUG was used (0.7%). This prevalence was much lower when compared with previous studies (2.5–21.6%) [42–44]. A possible explanation for the divergence in the prevalence rates across the studies refers to the cutoff point used for TUG performance. In this study, we used a cutoff of ≥ 20 s as recommended by the EWGSOP in 2019 [1], which is relatively high when compared with the cutoffs used by Paula et al. (2016) (> 11.3 s), Sui et al. (2021) (> 9.3 s), and Alexandre et al. (2012) (> 12.47 s) [42–44].

Despite the relevance of the findings of the present study, they should be considered with caution due to certain limitations. Firstly, the sample was obtained by convenience. Secondly, only women were included in this study, which meant that sex-related differences could not be evaluated. It would therefore be interesting to expand the study to include older men. Thirdly, our sample was exclusively composed of older women residing in a municipality of Brazil’s southeast region, which prevents extrapolating the results to populations of places with different sociodemographic and environmental characteristics.

Recent evidence highlights the need for a globally accepted definition of sarcopenia, as well as the need for operational parameters to better diagnose the disease [36, 45]. In this sense, our results add to existing knowledge by revealing the necessity for additional studies aimed at comparing diagnostic instruments within other existing consensuses and verifying the agreement between the available methods. Although the best instrument or criterion for diagnosing the presence of probable, confirmed, and severe sarcopenia is not yet known, researchers and health professionals should be aware of the differences in these definitions and their prevalence rates when applying the different instruments in older populations.

## Conclusion

There were differences in the prevalence rates of sarcopenia and low agreement between the diagnostic instruments proposed by the EWGSOP2. The findings of this study suggest that these issues must be considered in the discussion on the concept and assessment of sarcopenia, which could ultimately help to better identify patients with this disease in different populations.

## Data Availability

The datasets used and/or analyzed during the current study are available from the corresponding author upon reasonable request.
